# Commentary: Efficacy and safety of Xiaoyao San in the treatment of chronic fatigue syndrome: a systematic review and meta-analysis

**DOI:** 10.3389/fphar.2025.1597672

**Published:** 2025-08-18

**Authors:** Runhua Zhang, Hongxia Hu

**Affiliations:** ^1^ Department of Traditional Chinese Medicine, Wuwei Liangzhou Hospital, Wuwei, Gansu, China; ^2^ Department of Thoracic Surgery, Gansu Provincial Hospital, Lanzhou, Gansu, China

**Keywords:** Xiaoyao San, chronic fatigue syndrome, systematic review and meta-analysis, methodological bias, modified herbal interventions

We read with great interest the recent systematic review and meta-analysis evaluating the efficacy and safety of XYS for the treatment of CFS. This study provides timely and valuable insights, particularly in the context of the post-COVID era where CFS-like symptoms have received renewed attention. Synthesizing data from six randomized controlled trials (RCTs), the authors report that XYS significantly improves clinical symptoms such as fatigue, anxiety, and depression with few adverse events (Wang et al., 2025). While the findings are encouraging, several methodological and clinical issues require deeper reflection to assess the reliability, applicability, and future direction of this research.

First, we note significant limitations in the quality and standardization of the included RCTs. Most studies lacked clear reporting of key methodological areas, including allocation concealment and blinding of participants and outcome assessors. The Cochrane Risk of Bias tool identified several trials at high or unclear risk of overestimating treatment effects due to performance and detection bias. Importantly, none of the included trials used a double-blind design - an omission of particular concern in trials with subjective outcomes such as fatigue and mood symptoms. As previous literature suggests, lack of blinding may inflate effect sizes in trials evaluating complementary therapies (Whiting et al., 2001). Future trials should ensure methodological rigor by implementing appropriate randomization, allocation concealment, and blinding protocols where feasible.

Second, we are concerned about the substantial clinical and pharmacological heterogeneity introduced by the use of “Modified XYS” across the included trials. Although all formulations are based on the classical Xiaoyao San prescription, TCM syndrome differentiation often leads to additions or subtractions of herbs to address specific pathophysiological patterns. For instance, in cases of prominent Qi-deficiency, herbs such as Huang Qi may be added to strengthen vitality, while blood stasis patterns might prompt the inclusion of Dan Shen to invigorate circulation. These adjustments are not merely cosmetic—they can significantly alter the pharmacodynamic properties of the formula, influencing anti-inflammatory, neuroendocrine, or immunoregulatory effects. Consequently, pooling trials that used different Modified XYS formulations risks conflating distinct therapeutic interventions under a single effect estimate. Previous meta-analyses of TCM formulas have cautioned against this practice when core compositions differ meaningfully. A more refined classification distinguishing between shared core components and divergent adjunctive herbs could enhance interpretability and may reveal formulation-specific efficacy patterns.

Third, the use of “effective rate” (ER) as the primary outcome deserves critical scrutiny. ER is a composite endpoint commonly used in Chinese clinical trials, typically based on reductions in symptom scores beyond arbitrary thresholds. However, this metric lacks international standardisation and transparency, and critically, it fails to disaggregate which specific symptoms are driving the observed “effect.” In the context of CFS—a heterogeneous disorder characterised by core symptoms like debilitating fatigue alongside ancillary symptoms such as anxiety, insomnia, and cognitive impairment—this is a major limitation. For example, a participant who experiences mild relief of sleep disturbance but no improvement in fatigue may still be classified as “effective,” thereby inflating perceived treatment benefits. Moreover, ER does not capture the severity, persistence, or multidimensional nature of fatigue, which is central to the clinical burden of CFS. In contrast, validated instruments such as the Chalder Fatigue Scale quantify fatigue across both physical and mental domains (Chalder et al., 1993), while the SF-36 includes subscales for vitality, role functioning, and general health perceptions (Ware and Sherbourne, 1992). These tools enable a more nuanced, reproducible, and internationally comparable assessment of therapeutic outcomes. Future trials should prioritise such validated scales to enhance both the internal validity and global relevance of outcome measurement in CFS research.

Finally, while the review briefly outlines several possible mechanisms—such as modulation of mitochondrial energy metabolism, Th1/Th2 and Th17/Treg immune balance, HPA axis regulation, and gut microbiota composition—these remain largely speculative. To make future research more actionable, we recommend anchoring mechanistic exploration to key bioactive components of XYS. For example, Chai Hu, a principal herb in XYS, has been shown to modulate the hypothalamic–pituitary–adrenal axis by influencing corticotropin-releasing hormone and glucocorticoid receptor signalling (Li et al., 2017). Similarly, Dang Gui contains phthalides and polysaccharides that have demonstrated immunomodulatory and microbiota-altering effects in preclinical models (Zou et al., 2023). These mechanistic pathways align with emerging evidence of neuroendocrine and immunometabolic dysfunction in CFS pathophysiology. A systems biology framework—integrating metabolomics, microbiome profiling, and immune phenotyping—could thus illuminate which patient subgroups respond to XYS and why. This approach may also facilitate biomarker discovery for clinical monitoring and precision targeting.

In conclusion, this review contributes to the growing interest in XYS as a potential complementary therapy for CFS, but also underscores the urgent need for higher quality evidence. We recommend that future studies adopt standardized diagnostic criteria, employ validated outcome measures, ensure methodological rigor, and extend follow-up to assess sustainability and safety. In addition, mechanistic studies should aim to bridge preclinical findings with clinical phenotypes using integrated bioinformatics approaches. Only through such improvements can XYS be confidently positioned in the evidence-based management of CFS.
